# Prevention of postamputation pain with targeted muscle reinnervation (PreventPAP trial): protocol for a national, multicentre, randomised, sham-controlled trial

**DOI:** 10.1136/bmjopen-2025-105053

**Published:** 2025-11-04

**Authors:** Guus A H Tendijck, Jan van Schaik, Robert R Dijkman, Marieke Niesters, Erik W van Zwet, Wilbert B van den Hout, Arianne J Ploeg, Willem G van Rijt, Godard C W de Ruiter, J Henk Coert, Liron S Duraku, J Michiel Zuidam, Willemien van de Water, Willem Pondaag, Hanneke van der Krogt, Justus L Groen, PreventPAP consortium, Pieter S Verduijn

**Affiliations:** 1 Neurosurgery, Leiden University Medical Center, Leiden, The Netherlands; 2 Surgery, Leiden University Medical Center, Leiden, The Netherlands; 3 Plastic, Reconstructive and Hand Surgery, Leiden University Medical Center, Leiden, The Netherlands; 4 Anesthesiology and Pain Medicine, Leiden University Medical Center, Leiden, The Netherlands; 5 Biomedical Data Sciences, Leiden University Medical Center, Leiden, The Netherlands; 6 Surgery, Alrijne Zorggroep, Leiderdorp, The Netherlands; 7 Plastic, Reconstructive and Hand Surgery, Isala Hospital, Zwolle, The Netherlands; 8 Neurosurgery, Medisch Centrum Haaglanden, Den Haag, The Netherlands; 9 Plastic, Reconstructive and Hand Surgery, University Medical Centre Utrecht, Utrecht, The Netherlands; 10 Plastic, Reconstructive and Hand Surgery, Amsterdam University Medical Centres, Amsterdam, The Netherlands; 11 Plastic, Reconstructive and Hand Surgery, Erasmus MC, Rotterdam, The Netherlands; 12 Surgery, Maastricht UMC+, Maastricht, The Netherlands; 13 Neurosurgery, Alrijne Zorggroep, Leiderdorp, The Netherlands; 14 Rehabilitation Medicine, Leiden University Medical Center, Leiden, The Netherlands

**Keywords:** Amputation, Surgical, NEUROSURGERY, Clinical Protocols, PAIN MANAGEMENT, Randomized Controlled Trial, Vascular surgery

## Abstract

**Introduction:**

In the Netherlands, approximately 2200 major amputations of the lower extremities are performed each year, the majority in vascular patients. Around 61% of these patients will develop postamputation pain (PAP). PAP is a severe, lifelong, disabling condition profoundly affecting quality of life. During amputations, the common practice is to cut the nerves without employing nerve-surgical techniques to prevent chronic pain due to neuroma formation. In recent years, targeted muscle reinnervation (TMR) has been the most frequently studied technique for treating PAP, inhibiting neuroma formation by rerouting the cut mixed nerve to a functional motor nerve. We hypothesise that a primary TMR procedure during major lower limb amputations will result in a lower prevalence of PAP.

**Methods and analysis:**

We propose a national, multicentre, randomised, sham-controlled trial comparing TMR with traction neurectomy in major amputations of the lower extremities in patients with vascular disease. 203 patients will be recruited with an indication for a transfemoral to transtibial amputation as a primary or secondary sequela of vascular disease. The subjects are randomly assigned to the TMR group or the traction neurectomy group. PAP will be evaluated 1 year postoperatively as the primary endpoint. Secondary outcomes include quality of life, mobility, neuropathic pain, hospital anxiety and depression, cost-effectiveness and complications.

**Ethics and dissemination:**

This study has been reviewed and approved by the local ethical review body, ‘The Medical Ethics Committee Leiden The Hague Delft’, under the reference: P24.073 on 28 November 2024. Results will be published in peer-reviewed journals.

**Trial registration number:**

NCT06719245. Dutch trial registry: NL87196.058.24

**STRENGTHS AND LIMITATIONS OF THIS STUDY**

- The multicentre, randomised, sham-controlled design minimises potential bias.
- The study is appropriately powered to detect differences in both phantom limb pain and residual limb pain.
- The use of multiple validated questionnaires, including patient-reported outcome measures, ensures robust assessment of the treatment effect.
- The expected inclusion is limited to 40% of eligible patients due to the availability of nerve surgeons and the additional operating time in a semiacute setting.

## Introduction

### Background and rationale

 Approximately 2200 major lower extremity amputations, transfemoral to transtibial, are performed in the Netherlands each year, and the incidence is rising, particularly due to vascular disease.^1 2^ Around 61% of these patients develop postamputation pain (PAP).^3^ PAP is known to compromise prosthetic rehabilitation and profoundly diminish the quality of life after amputation.^4^ Patients with chronic pain report a lower health-related quality of life when a neuropathic component is present (0.70 vs 0.47).^5^ Moreover, it is associated with a significant burden on the healthcare system.^6^ Direct healthcare costs may comprise prehospital care, emergency care, hospitalisation, long-term postdischarge care and rehabilitation (eg, increased opioid use and other (neuropathic) pain medication and interventions by pain specialists to treat PAP). Indirect costs may arise as a consequence of loss of productivity for both patients and their families.

PAP falls into two major categories: residual limb pain (RLP, ‘stump pain’) and phantom limb pain (PLP). RLP has different aetiologies, including infection, vascular insufficiency, scar tissue, bone spurs and neuroma formation.^7^ PLP is caused by transection of the nerves during amputation. While the exact mechanism behind PLP is not yet fully understood, it is partly explained by deafferentation from the distal target. Currently, there is no uniform protocol for the surgical handling of nerves during amputations. There is little attention for nerve handling in surgical textbooks, the Dutch Guideline ‘Amputation and prosthetics of the lower extremity’ and the Global Vascular Guideline on the Management of Chronic Limb-Threatening Ischaemia do not mention nerve handling during amputation surgery.^4 8^ Today, techniques used depend primarily on traditions and expert opinions, which lack a solid scientific base. Nerve handling is performed with sharp transection or electrocautery, often using light traction or ligation. After the nerve is cut, it may be tucked away, coagulated, infiltrated with a local anaesthetic or alcohol or it may be left untreated.^9^

When PAP develops, it is very challenging to treat.^10^ If non-surgical treatments fail or side effects of medication dominate a patient’s life, surgery for PAP may be performed. Nerve-surgical techniques that are then applied differ widely and, again, lack any solid scientific base. In general, secondary surgery for PAP includes resection of the neuroma and translocation of the freshened nerve into surrounding tissue, such as muscle, bone or fat, or more advanced techniques like targeted muscle reinnervation (TMR), regenerative peripheral nerve interface or nerve capping.^11^,^13^

Several cohort studies and a recent randomised trial show that the nerve-surgical technique TMR is effective in the reduction of PAP, with limited complications and improved quality of life.^14^,^16^ As persistent nociceptive stimulation in chronic pain may cause various changes in pain physiology, leading to pain sensitisation, preventing this chronic pain syndrome might gain even larger benefits. Few small non-randomised cohort studies are published on primary TMR during or shortly after amputations.^17^,^23^ Despite issues with the validity of the articles, results were consistently in favour of primary TMR relative to standard neurectomy regarding all outcome measures, including intensity and interference with daily activities. Although the pooled estimates in the meta-analysis should be interpreted with great caution because of between-study heterogeneity, the risk of RLP and PLP may be halved when performing TMR.

Moreover, Deeyor *et al*^18^ calculated the 1-year healthcare cost in patients undergoing standard amputation and in those receiving an amputation with primary TMR. The average healthcare costs for the primary TMR group were $32 632 versus $36 219 for those receiving standard care (Phoenix, USA), supporting the cost-effectiveness of the intervention.^18^

The main drawbacks of TMR are that it is a nerve-surgical technique not routinely performed by amputation surgeons and that it is a time-consuming procedure, which might be cumbersome, especially in the semiacute setting of amputation surgery.

### Objective and hypothesis

In this study, we aim to compare primary TMR with traction neurectomy during a transfemoral to transtibial amputation as a primary or secondary sequela of vascular disease. We hypothesise that primary TMR will decrease PAP and improve quality of life at lower costs.

## Methods

### Study design

The PreventPAP trial investigators propose a national, multicentre, randomised, single-blind, sham-controlled trial comparing primary TMR with traction neurectomy in patients who undergo transtibial to transfemoral amputations as a primary or secondary sequela of vascular disease. The protocol is developed in accordance with the Standard Protocol Items: Recommendations for Interventional Trials guidelines.^24^ A summary of the trial information is presented according to the WHO Trial Registration Data Set (online supplemental file 1).

### Study population

The study population includes patients who receive a major amputation of the lower extremities as a primary or secondary sequela of vascular disease. To ensure a homogenous cohort, only patients with an amputation as a result of vascular pathology are included. In the Netherlands, this group accounts for 90%–95% of all amputations of the lower extremities.^4^ Moreover, as age above 75 years is a major risk factor for 1-year mortality, this group was excluded.^25^

Inclusion criteria

- Patients aged between 18 and 75 years.
- Scheduled for a transtibial, through-knee or transfemoral amputation as a primary or secondary sequela of vascular disease.

Exclusion criteria

- Insensate limbs at the level of amputation.
- Complex Regional Pain Syndrome.
- Existing neuroma or prior neuroma surgery in the affected limb.
- Undergoing radiotherapy on the affected limb.
- Cognitive impairment or delirium at the time of consent.
- Patients who are unfit for general anaesthesia.
- No nerve surgeon trained in the TMR procedure is available.

### Recruitment and consent

203 patients undergoing a major lower limb amputation will be randomised into two equal parallel groups after informed consent is given. One group will receive TMR, and the other group will receive traction neurectomy. Patients will be recruited from academic and non-academic hospitals in the Netherlands via the emergency department, ward or the outpatient clinic. After the indication for amputation is set, eligibility for inclusion is evaluated by the treating physician. Afterwards, the physician will contact an authorised local investigator to discuss the trial, obtain written informed consent (online supplemental file 2) and randomise the patient.

Patients who have undergone a major leg amputation and were not included in the trial will be consented for a parallel prospective registry study. This registry study is part of the PreventPAP trial and will be used as external validation and to enhance the interpretation of the trial results. Including all patients undergoing amputation could expose selection bias and make this study more pragmatic. In this study, we plan to report the same baseline, intraoperative and postoperative variables, and we will send the same questionnaires at 12 months, except for the cost-effectiveness questionnaires. This allows us to compare the outcome between the randomised and non-randomised groups, providing additional context and validation of the trial results.

### Study procedures

After randomisation, patients will receive general anaesthesia without a muscle relaxant for the TMR group. Intraoperative epidural anaesthesia is not allowed as this will reduce nerve conduction, making nerve stimulation less dependable. The amputation will be performed by a qualified vascular surgeon or a senior resident in training under supervision. The skin will be incised using a fishmouth incision (transfemoral and through-knee) and according to Burgess’ technique (transtibial). If patients are randomised in the TMR group, a trained nerve surgeon will perform the nerve coaptation. Postoperative protocol is similar for both interventions, including the use of analgesia. In transfemoral and through-knee amputations, there will be an additional 10-centimetre incision on the posterior side of the leg. This additional incision is necessary to coaptate the sciatic nerve. To ensure proper blinding, a sham skin incision will be performed in the control group. Closure and dressing of the wound will be similar between groups.

To ensure homogeneity in all participating centres a treatment and postoperative care protocol will be distributed for both the intervention and control groups (online supplemental file 3), in addition to a central cadaver-based TMR course.

#### Control group

The patients in the control group will receive traction neurectomy during the major lower limb amputation, which is the current standard of care in the Netherlands, and this will reduce confounding by the length of the nerve stumps. Ligation or infiltration with a local anaesthetic or alcohol will not be allowed, as we believe this causes significantly more PAP. Amputation technique and nerve handling are carefully documented according to standardised protocols.

#### TMR group

**Figure 1:**
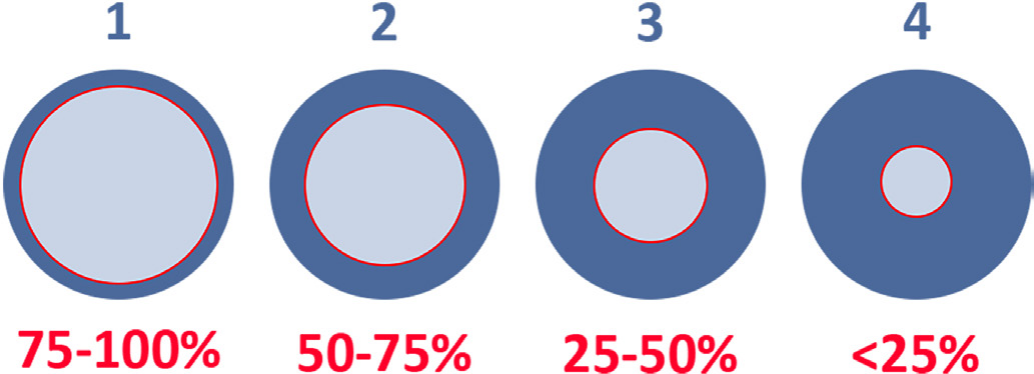
Nerve coaptation calibre mismatch score. Outer circle: amputated nerve; inner circle: motor nerve.

The patients in the TMR group will receive the same amputation technique as the control group, with an additional TMR procedure. TMR will be performed by an experienced nerve surgeon. In the TMR group, each transected nerve identified after amputation is dissected proximally for length. A nerve stimulator is used to identify functional motor nerve branches near the point where the motor branch enters the muscle. The motor nerve branch is transected proximally, and an end-to-end coaptation is performed with the amputated nerve. A major point of criticism in TMR is the size mismatch between the larger amputated nerve and the smaller motor nerve during coaptation. To better understand this factor, a nerve coaptation calibre mismatch score was used in an attempt to standardise the measurement (figure 1).

There will not be an expected treatment delay in the TMR group; however, the operation will take approximately 30–90 min longer.^17^ For TMR to be possible in upper leg amputations, an additional incision (around 10 cm) has to be made on the dorsal side of the leg, medial to the sartorius muscle, to coaptate the sciatic nerve. If a tourniquet is used to perform the amputation, the maximum time of inflation is 60 min, after which intraoperative nerve stimulation becomes less reliable due to tourniquet neuropraxia.

### Outcomes

Longitudinal studies show no further improvement of PAP after TMR in amputees after 12 months postoperatively.^15 23 26 27^ Therefore, our follow-up time will be 12 months. Outcomes will be assessed at 2 weeks and at 3, 6, 9 and 12 months via questionnaires (table 1). These will be completed digitally via email, using a pseudo-anonymised data management platform. Patients allocated to the control group who experience significant pain at the end of the study will be offered additional TMR, regardless of amputation level.

**Table 1:** Schedule of enrolment PreventPAP trial

Study period	Enrolment	Allocation	Postallocation						Close-out
Timepoint in Months	0	0	0.5	3	6	9	11	12	12
Enrolment:									
Screening/eligibility criteria	X								
Informed consent	X								
Allocation		X							
Assessment:									
Baseline characteristics	X	X							
Preoperative/intraoperative/postoperative variables	X	X							
Questionnaires:									
NRS RLP and PLP*				X	X	X	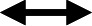	X†	
PROMIS Pain Behaviour 7a Short Form^29^				X	X	X		X†	
PROMIS Pain Interference 8a Short Form^29^				X	X	X		X†	
EQ-5D-5L^32^			X	X	X	X		X	
ICAN Pain Sketches^30^				X	X	X		X	
iMCQ and iPCQ^36 37^				X	X	X		X	
PainDetect^31^								X	
HADS^33^								X	
GPE-DV^34^								X	
PLUS-M^35^								X	
Close-out:									
Outcome									X
Medication‡		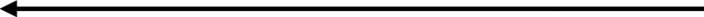							

* NRS for RLP and PLP will be measured every day for 30 consecutive days at 11 months postamputation.

† Primary endpoint.

‡ Medication will be recorded at the end of the study for the whole study period.

EQ-5D-5L, European Quality of Life - 5 Dimensions - 5 Levels; GPE-DV, Global Perceived Effect - Dutch Version; HADS, Hospital Anxiety and Depression Scale; ICAN, Interdisciplinary Care for Amputee Network; iMCQ, Medical Consumption Questionnaire; iPCQ, Productivity Cost Questionnaire; NRS, Numeric Rating Scale; PLP, phantom limb pain; PLUS-M, Prosthetic Limb Users Survey of Mobility; PROMIS, Patient-Reported Outcomes Measurement Information System; RLP, residual limb pain.

#### Main study parameter/endpoint

The primary endpoint will be pain experience 1 year postoperatively. This will be scored for RLP and PLP on an 11-point (0–10) Numeric Rating Scale (NRS) for 30 consecutive days and averaged. Patients will receive additional textual and visual information on how to differentiate between RLP and PLP in the questionnaires. We will use the Medication Quantification Scale to correct for pain medication use.^28^ Besides the NRS, pain will also be scored using the Patient-Reported Outcomes Measurement Information System (PROMIS) pain behaviour and interference short forms (7a and 8a, respectively) in Dutch.^29^

#### Secondary study parameters/endpoints

Secondary endpoints include the use of analgesics or pain interventions, surgical time, length of hospital stay and adverse events or complications, including survival. In addition, multiple questionnaires will be sent to the participants regarding RLP and PLP during the study (measured by the NRS); pain behaviour and pain interference during the study (measured by the PROMIS Pain Behaviour and Pain Interference Short Forms)^29^; local, diffuse or radiating pain (measured by the Interdisciplinary Care for Amputee Network pain sketches)^30^; neuropathic pain (measured by the PainDetect)^31^; quality of life (measured by the European Quality of Life - 5 Dimensions - 5 Levels (EQ-5D-5L))^32^; anxiety and depression symptoms (measured by the Hospital Anxiety and Depression Scale)^33^; perceived treatment effect (measured by the Global Perceived Effect - Dutch Version)^34^ and mobility 1 year after surgery (measured by the Prosthetic Limb Users Survey of Mobility).^35^ A cost-effectiveness analysis (CEA; measured by the Medical Consumption Questionnaire (iMCQ))^36^ and the Productivity Cost Questionnaire (iPCQ)^37^ and a budget impact analysis (BIA) (measured by the ZonMw BIA tool) will also be performed at the end of the study. All data will be collected using validated questionnaires at different time points (table 1).

### Data collection and data management

By embracing the Findability, Accessibility, Interoperability and Reusability of digital assets principles, we underscore our commitment to robust, transparent and ethically sound scientific practices that advance research integrity and propel scientific progress. Using the Leiden University Medical Centre (LUMC) Data Management Tool, with the support of the LUMC section of Advanced Data Management, we ensure that our data are findable, enabling easy discovery through well-structured metadata and standardised identifiers. Through our commitment to accessibility, we guarantee that both researchers and the broader community can access our data with minimal barriers, fostering collaboration and knowledge dissemination. Interoperability remains a focal point, as we structure our data in standardised formats and use established vocabularies, facilitating seamless integration with other datasets and tools. Our dedication to reusability ensures that the data generated through our trial can be leveraged beyond its initial purpose, promoting long-term scientific impact and innovation.

Data collection is done in standardised electronic databases. All relevant clinical data will be entered into electronic Case Report Forms. Data storage and backup will be managed by the Castor Electronic Data Capture (EDC) system. Data will be managed confidentially and coded in compliance with the European Union General Data Protection Regulation. Castor’s encryption module will be used for pseudonymisation. All analyses will be performed on de-identified, pseudonymised, coded data, for which explicit permission is given in the patient informed consent form. A subject identification code list will be used to link the data to each subject when needed for data collection. Once assigned, the number will not be reused if the patient is excluded. After completion of the study, the key file will be archived in the hospital’s study documentation in a protected location on the network hard drive for 15 years in accordance with Article 17 of the European Good Clinical Practice Directive.

The LUMC is the trial sponsor and will have access to the final dataset. A clinical trial agreement is distributed among the other participating centres.

### Sample size calculation

**Figure 2:**
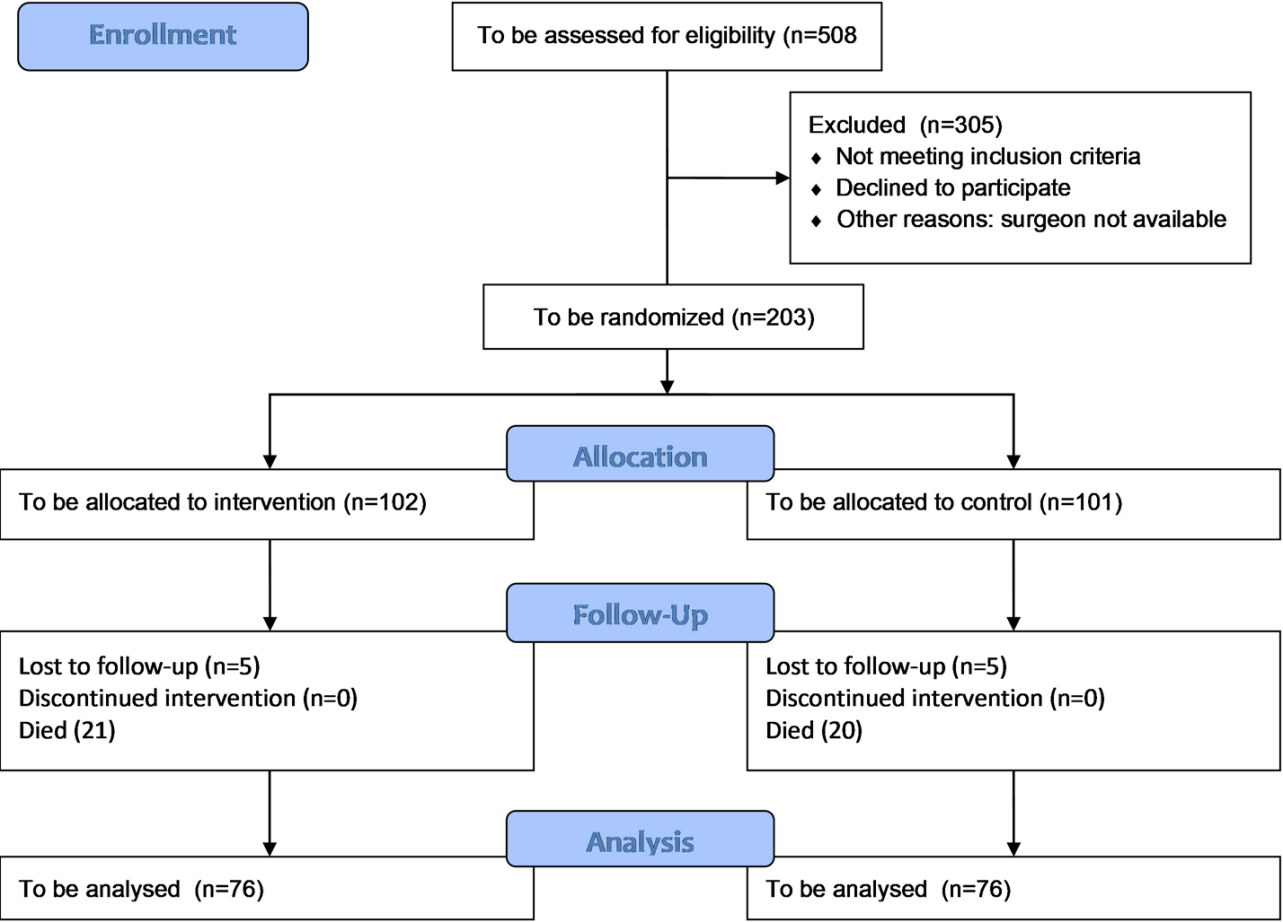
Consolidated Standards of Reporting Trials (CONSORT) flow diagram of predicted patient inclusion.

In studies of both chronic and acute pain, a change of two points on the NRS has been shown to be clinically relevant and correlated to a patient’s need to take additional pain medication.^38^ To detect a minimal clinically important difference of two points on the NRS, considering an SD of 3.5 from similar studies that measured pain at one time point after 12 months^15 23 26 27^ in a clinically heterogeneous group, with a significance level of 0.025, we need to analyse 2×76=152 patients to achieve 90% power. With an estimated loss to follow-up of 6%, we need to include 162 patients. Moreover, based on the study by Cascini *et al*^25^ and our clinical experience, we expect a 1-year mortality rate of 20% in our patient population aged 75 years or younger. With an estimated 1-year survival of this patient group of 80%, we need to include 203 patients in total. Due to the semiacute setting of most amputations, organising the availability of both extra operating time and a trained nerve surgeon might be a challenge. Therefore, we estimate including 40% of all major lower extremity amputations. To be able to randomise 203 subjects, we estimate the need to screen 508 patients (figure 2).

### Randomisation, blinding and treatment allocation

The Randomisation Module within Castor EDC will be used to assign participants to specific groups (web-based randomisation/treatment allocation system). In this way, an unpredictable allocation sequence will be generated so that the treatment allocation is concealed from the patient, nurses and rehabilitation professionals. Simple randomisation is used to reduce the risk of selection and accidental bias. Randomisation will not be stratified by centre because of the risk of selection bias due to treatment predictability in centres with low inclusion rates. Furthermore, due to the anticipated low inclusion rate in some centres, an imbalance in the number of patients per study arm can be created when working with stratification by centre. Therefore, randomisation will be global over all participating centres. Patients will be blinded to the allocation until the end of the study, 1 year after the last patient is included.

### Statistical analysis

Continuous variables will be summarised by treatment group using the number of non-missing data points, mean, SD, median and IQR. Categorical and ordinal variables will be summarised by treatment group based on observed frequencies and percentages relative to the total number of non-missing items. To account for the correlation between measurements within the same patient, a repeated measures analysis will be conducted. This will allow us to include all participants with at least a single follow-up measurement. Where appropriate, we will include age, sex and amputation level in our analyses.

#### Primary study parameter(s)

We selected four co-primary endpoints on the basis of the expected impact of the intervention and relevance for the patients. They are all primarily assessed at 12 months after surgery:

1. RLP (NRS).
2. PLP (NRS).
3. PROMIS Pain Behaviour Short Form (7 a).
4. PROMIS Pain Interference Short Form (8 a).

These outcomes will be compared between the two treatment groups on the basis of the intention-to-treat principle. The comparisons will be adjusted for age, sex and amputation level, based on clinical relevance. To account for multiple comparisons, we proceed as follows. We start by testing outcomes 1 and 2 at a significance level of 0.025 (two-sided) using the Bonferroni correction. This is the basis of our sample size calculation. Next, we apply a serial gatekeeping procedure:

- If both outcomes 1 and 2 reach statistical significance, outcome 3 will be tested at a significance level of 0.05 (two-sided). If outcome 3 reaches statistical significance at that level, outcome 4 will be tested at a significance level of 0.05 (two-sided).
- If either outcome 1 or 2 reaches statistical significance, outcome 3 will be tested at a significance level of 0.025 (two-sided). If outcome 3 reaches statistical significance at that level, outcome 4 will be tested at a significance level of 0.025 (two-sided).^39^

#### Secondary study parameters

All secondary study parameters will be compared using the appropriate tests, which are subsequently adjusted for age, sex and amputation level. Categorical variables will be analysed using the χ^2^ test or Fisher’s exact test and adjusted with a logistic regression model. Continuous variables will be assessed using the Student’s t-test or Mann-Whitney U test and adjusted using a linear regression model.

#### Cost-effectiveness analysis (CEA)

The economic evaluation will be a trial-based cost-utility analysis (CUA) from a societal perspective (CUA, ie, costs per quality-adjusted life years (QALYs)). Care with and without TMR will be compared with a 1-year time horizon. Estimated societal costs will include hospital (based on study registrations) and other healthcare and productivity costs, using the iMCQ^36^ and iPCQ^37^ at 3, 6, 9 and 12 months. A cost-price analysis will be performed for the TMR. Other healthcare costs will be valued according to the Dutch reference prices whenever possible, including travel costs without discounting (because of the short time horizon). Productivity losses will be valued using the friction-cost method. QALYs will be calculated using the Dutch tariff for the EQ-5D-5L,^32^ at 2 weeks and at 3, 6, 9 and 12 months. Average costs and QALYs will be compared according to ITT, using net-benefit analysis and multiple imputation to account for missing data. Sensitivity analyses will include the cost perspective (healthcare instead of societal) and the utility measure (European Quality of Life visual analogue scale with power-transformation instead of the EQ-5D-5L). If, contrary to expectations, TMR does not deliver better QALYs at lower costs, mathematical extrapolation will be used to perform a threshold analysis on the time horizon.

#### Budget impact analysis (BIA)

A BIA will be performed using the ZonMw BIA tool to estimate the financial impact of different implementation scenarios at the national level. The analysis will be based on the costs as estimated during the study and the expected number of patients in the Netherlands. The BIA will be conducted from the perspectives of society, the hospital and ‘Budgettair Kader Zorg’ (translates to ‘budgetary framework for healthcare’), with the appropriate prices and charges. Costs will be estimated per budget period (1 year) for a time horizon of 5 years, assuming 50%–100% implementation after 4 years.

### Benefits and risk assessment, group relatedness

The additional risks of performing TMR during an amputation are negligible. TMR can be performed at any level of the lower extremity with a standardised technique. For TMR to be possible in transfemoral and through-knee amputations, an additional sham incision (around 10 cm) must be made on the dorsal side of the leg, medial to the sartorius muscle. To properly blind study participants, this additional incision must also be superficially performed for the transfemoral and through-knee amputations in the control group. In our experience, this will not result in increased postoperative pain or difficulty sitting.

The surgery time for a standard transtibial and transfemoral amputation will be 45–90 min, depending on the surgeon’s experience. The TMR procedure will add 30–90 min to the surgery.^17^ This extra time investment will rely on technical aspects related to the level of amputation and the surgeon’s experience. Although an increase in surgical time of this length is related to a slightly higher risk of infection, Deeyor *et al*^18^ did not find more complications in patients who had acute TMR compared with the standard of care. Deeyor *et al* found a 77% complication rate in the primary TMR group, compared with 87% in the amputation-only group.^18^ There is a small chance of failure of the TMR procedure.^40^ If patients suffer harm from trial participation, the LUMC provides participant insurance for potential financial compensation.

The burden of the study is acceptable, as participation only requires patients to complete several questionnaires at five evaluation time points. This will translate to about 2 min at 2 weeks, 14–21 min at 3, 6 and 9 months and 20–29 min at 12 months postamputation. Additionally, participants will fill out a daily questionnaire consisting of 2 questions about pain for 30 consecutive days at 11 months postamputation. Patients who fail to complete the questionnaires in 2 weeks will receive an automatic reminder and a call from the researcher to promote participant retention.

Depending on the literature source and the level of amputation, the frequency of PAP ranges between 33% and 86%.^3^ In the patient series performed on primary TMR, PAP was present in 8%–29%.^1719^,^22^ After primary TMR, 90.9% of patients who had TMR were ambulatory compared with 70.5% (p<0.01) in the amputation-only group. O’Brien *et al* showed a longitudinal durability of this effect over 18 months or more in 81 patients.^23^ One trial on TMR as a secondary procedure^14^ showed an average decrease in pain NRS of 3.2 in the TMR arm compared with an average increase of 0.2 in the standard treatment arm. Various cohort studies on secondary TMR show similar results.

Primary TMR results in a reduction of the chance of developing PAP. The risks and the burden for patients are negligible.

Because the risks of TMR are negligible, no data safety management board has been assigned. During the study, patient safety and execution of study procedures will be closely monitored by an independent clinical trial monitor and auditor according to local regulations.

### Ethics and dissemination

The local medical ethics committee of Leiden The Hague Delft approved the study (reference number P24.073). Protocol amendments and adverse events during the study will be reported and evaluated by the local ethics committee and communicated with the participating centres if needed. Before randomising patients, an informed consent form must be signed.

The results of the study will be presented at both international and national conferences and published in peer-reviewed journals. Moreover, results will also be disseminated to the public using the Dutch patient association for amputees, ‘KorterMaarKrachtig’.

### Patient and public involvement

The Dutch patient association for patients with an amputation, ‘KorterMaarKrachtig’, was involved in the development of the study. The primary outcome measures were discussed and jointly decided. Also, they assisted with the development of patient information. Together, we developed intelligible patient information and provided participants with understandable information about the goals, methodology and the potential risks and benefits of the trial.

To inform patients about the developments during the trial, we will provide updates two times a year in their journal, present at patient days and provide access to the study results once available. Moreover, the results will be discussed with the patient association, and they will be involved in further research plans on this subject and the implementation of this technique in common practice.

## Supplementary material

10.1136/bmjopen-2025-105053 online supplemental file 1

10.1136/bmjopen-2025-105053 online supplemental file 2

10.1136/bmjopen-2025-105053 online supplemental file 3

## References

[1] Frölke JPM, Rommers GMC, de Boer AW (2024). Epidemiology of Limb Amputations and Prosthetic Use During COVID-19 Pandemic in the Netherlands. Arch Phys Med Rehabil.

[2] Ziegler-Graham K, MacKenzie EJ, Ephraim PL (2008). Estimating the prevalence of limb loss in the United States: 2005 to 2050. Arch Phys Med Rehabil.

[3] Schwingler PM, Moman RN, Hunt C (2021). Prevalence of postamputation pain and its subtypes: a meta-analysis with meta-regression. Pain Rep.

[4] Nederlandse Vereniging van Revalidatieartsen (2020). Amputatie en prothesiologie onderste extremiteit. https://richtlijnendatabase.nl/richtlijn/amputatie_prothesiologie_onderste_extremiteit/startpagina_-_amputatie_en_prothesiologie_onderste_extremiteit.html.

[5] Torrance N, Lawson KD, Afolabi E (2014). Estimating the burden of disease in chronic pain with and without neuropathic characteristics: does the choice between the EQ-5D and SF-6D matter?. Pain.

[6] Parsons B, Schaefer C, Mann R (2013). Economic and humanistic burden of post-trauma and post-surgical neuropathic pain among adults in the United States. J Pain Res.

[7] Liu K, Tang T, Wang A (2015). Surgical revision for stump problems after traumatic above-ankle amputations of the lower extremity. BMC Musculoskelet Disord.

[8] Conte MS, Bradbury AW, Kolh P (2019). Global vascular guidelines on the management of chronic limb-threatening ischemia. J Vasc Surg.

[9] de Bruijn ME, Arts CH, van de Meent H (2020). Management of the sciatic nerve during transfemoral amputation: a survey of Dutch surgeons. J Cardiovasc Surg (Torino).

[10] Poyntz SA, Hacking NM, Dalal M (2018). Peripheral Interventions for Painful Stump Neuromas of the Lower Limb: A Systematic Review. Clin J Pain.

[11] Ives GC, Kung TA, Nghiem BT (2018). Current State of the Surgical Treatment of Terminal Neuromas. Neurosurgery.

[12] Dellon AL, Mackinnon SE (1986). Treatment of the painful neuroma by neuroma resection and muscle implantation. Plast Reconstr Surg.

[13] Chou J, Liston JM, DeGeorge BR (2023). Traditional Neuroma Management Strategies: A Systematic Review. Ann Plast Surg.

[14] Dumanian GA, Potter BK, Mioton LM (2019). Targeted Muscle Reinnervation Treats Neuroma and Phantom Pain in Major Limb Amputees: A Randomized Clinical Trial. Ann Surg.

[15] Berger LE, Shin S, Haffner ZK (2023). The application of targeted muscle reinnervation in lower extremity amputations: A systematic review. Microsurgery.

[16] Walsh AR, Lu J, Rodriguez E (2023). The Current State of Targeted Muscle Reinnervation: A Systematic Review. J Reconstr Microsurg.

[17] Chang BL, Hill AL, Mondshine J (2024). Primary Targeted Muscle Reinnervation in Above-Knee Amputations in Patients with Unsalvageable Limbs from Limb-Threatening Ischemia or Infection. J Reconstr Microsurg.

[18] Deeyor ST, Kisana HM, Hui CH (2022). Targeted Muscle Reinnervation Does Not Increase the Risk of Postsurgical Complication or Overall Cost. Plast Reconstr Surg Glob Open.

[19] Frantz TL, Everhart JS, West JM (2020). Targeted Muscle Reinnervation at the Time of Major Limb Amputation in Traumatic Amputees: Early Experience of an Effective Treatment Strategy to Improve Pain. JB JS Open Access.

[20] Valerio IL, Dumanian GA, Jordan SW (2019). Preemptive Treatment of Phantom and Residual Limb Pain with Targeted Muscle Reinnervation at the Time of Major Limb Amputation. J Am Coll Surg.

[21] Alexander JH, Jordan SW, West JM (2019). Targeted muscle reinnervation in oncologic amputees: Early experience of a novel institutional protocol. J Surg Oncol.

[22] O’Brien AL, Jordan SW, West JM (2021). Targeted Muscle Reinnervation at the Time of Upper-Extremity Amputation for the Treatment of Pain Severity and Symptoms. J Hand Surg Am.

[23] O’Brien AL, West JM, Gokun Y (2022). Longitudinal Durability of Patient-Reported Pain Outcomes after Targeted Muscle Reinnervation at the Time of Major Limb Amputation. J Am Coll Surg.

[24] Chan A-W, Tetzlaff JM, Gøtzsche PC (2013). SPIRIT 2013 explanation and elaboration: guidance for protocols of clinical trials. BMJ.

[25] Cascini S, Agabiti N, Davoli M (2020). Survival and factors predicting mortality after major and minor lower-extremity amputations among patients with diabetes: a population-based study using health information systems. BMJ Open Diabetes Res Care.

[26] Mioton LM, Dumanian GA, Shah N (2020). Targeted Muscle Reinnervation Improves Residual Limb Pain, Phantom Limb Pain, and Limb Function: A Prospective Study of 33 Major Limb Amputees. Clin Orthop Relat Res.

[27] Kang NV, Woollard A, Michno DA (2022). A consecutive series of targeted muscle reinnervation (TMR) cases for relief of neuroma and phantom limb pain: UK perspective. J Plast Reconstr Aesthet Surg.

[28] Harden RN, Weinland SR, Remble TA (2005). Medication Quantification Scale Version III: Update in Medication Classes and Revised Detriment Weights by Survey of American Pain Society Physicians. J Pain.

[29] Terwee CB, Roorda LD, de Vet HCW (2014). Dutch-Flemish translation of 17 item banks from the patient-reported outcomes measurement information system (PROMIS). Qual Life Res.

[30] Gomez-Eslava B, Raasveld FV, Hoftiezer YAJ (2024). Pain Sketches to Predict Pain following Primary Targeted Muscle Reinnervation in Amputees. Plast Reconstr Surg.

[31] Freynhagen R, Baron R, Gockel U (2006). painDETECT: a new screening questionnaire to identify neuropathic components in patients with back pain. Curr Med Res Opin.

[32] Herdman M, Gudex C, Lloyd A (2011). Development and preliminary testing of the new five-level version of EQ-5D (EQ-5D-5L). Qual Life Res.

[33] Zigmond AS, Snaith RP (1983). The hospital anxiety and depression scale. Acta Psychiatr Scand.

[34] Hudak PL, Wright JG (2000). The Characteristics of Patient Satisfaction Measures. Spine (Phila Pa 1986).

[35] Anné C, Laat FA, Hafner BJ (2022). Dutch-flemish translation of the prosthetic limb users survey of mobility (PLUS-M).

[36] iMTA Productivity and Health Research Group (2018). Manual imta medical cost questionnaire (IMCQ).

[37] Bouwmans C, Krol M, Severens H (2015). The iMTA Productivity Cost Questionnaire: A Standardized Instrument for Measuring and Valuing Health-Related Productivity Losses. Value Health.

[38] Farrar JT, Young JP, LaMoreaux L (2001). Clinical importance of changes in chronic pain intensity measured on an 11-point numerical pain rating scale. Pain.

[39] Bretz F, Maurer W, Brannath W (2009). A graphical approach to sequentially rejective multiple test procedures. Stat Med.

[40] Felder JM, Pripotnev S, Ducic I (2022). Failed Targeted Muscle Reinnervation: Findings at Revision Surgery and Concepts for Success. Plast Reconstr Surg Glob Open.

