# Supplementary material for: Prevention of postamputation pain with targeted muscle reinnervation (PreventPAP trial): protocol for a national, multicentre, randomised, sham-controlled trial
Source: BMJ Open. 2025 Nov 4;15(11):e105053. doi: 10.1136/bmjopen-2025-105053 (PMC12587972; doi:10.1136/bmjopen-2025-105053)
Supplement: online supplemental file 1 [file bmjopen-15-11-s001.pdf]

Supplementary file 1. PreventPAP WHO trial registration data set

Data category	Information
Primary registry and trial identification number	ClinicalTrials.gov<br>NCT06719245
Date of registration in primary registry	7 December, 2024
Secondary identifying numbers	Dutch trial registry: NL87196.058.24<br>METC LDD: P24.073
Source(s) of monetary or material support	This project is funded via the Program ‘Potentially Promising Care’ by the National Health Care Institute and ZonMw
Primary sponsor	Leiden University Medical Center
Contact for public queries	Dr. J.L. Groen, MD Phd<br>Zenuwchirurgie@lumc.nl<br>+31715262109
Contact for scientific queries	Dr. J.L. Groen, MD Phd<br>j.l.groen@lumc.nl
Public title	Prevention of PostAmputation pain with Targeted Muscle Reinnervation
Scientific title	Prevention of PostAmputation pain with Targeted Muscle Reinnervation: a national, multicenter, randomized, sham-controlled trial
Countries of recruitment	Netherlands
Health condition(s) or problem(s) studied	Amputation pain in patients with a transfemoral to transtibial amputation due to vascular disease.
Intervention(s)	Control: standard neurectomy during an amputation, sham-controlled
	Intervention: Targeted Muscle Reinnervation during an amputation.
Key inclusion and exclusion criteria	Inclusion criteria:<br>- Patients aged between 18 and 75 years<br>- Scheduled for a transtibial, through-knee, or transfemoral amputation as a primary or secondary sequela of vascular disease.
	Exclusion criteria:<br>- Insensate limbs at the level of amputation<br>- Complex Regional Pain Syndrome<br>- Existing neuroma or prior neuroma surgery in the affected limb<br>- Undergoing radiotherapy on the affected limb<br>- Cognitive impairment or delirium at the time of consent<br>- Patients who are unfit for general anesthesia

	- No nerve surgeon trained in the TMR procedure is available
Study type	Interventional
	Allocation: randomized, single blind (subject, nurse, rehabilitation team, caregivers)
	Primary purpose: prevention
Date of first enrolment	31 December, 2024
Target sample size	203
Recruitment status	Recruiting at the time of submission
Primary outcome(s)	Postoperative pain, at 12 months postamputation using four co-primary endpoints:<br>1. Phantom limb pain (PLP), measured using the numeric rating scale (NRS) for 30 consecutive days and averaged.<br>2. Residual limb pain (RLP), measured using the NRS for 30 consecutive days and averaged<br>3. Patient Reported Outcome Measures Information System (PROMIS) pain behavior short form<br>4. PROMIS interference short form
Key secondary outcomes	- NRS for PLP and RLP, and PROMIS pain behavior and interference short forms at 3,6, and 9 months postamputation.<br>- Interdisciplinary Care for Amputees Network pain sketches at 3,6,9, and 12 months post amputation<br>- Quality of life measured using the EQ-5D-5L at 2 weeks, and 3,6,9, and 12 months postamputation<br>- PainDetect measured at 12 months postamputation<br>- Hospital anxiety and depression measured using the Hospital Anxiety and Depression Scale at 12 months postamputation<br>- Prosthetic rehabilitation measured using the Prosthetic Limb Users Survey of Mobility at 12 months postamputation<br>- Global perceived effect measured at 12 months postamputation<br>- Cost effectiveness measured using the Medical Consumption Questionnaire and the Productivity Costs Questionnaire<br>- Pain medication use and complications/adverse events.
Ethics Review	Leiden - Medical Ethics Committee Leiden The Hague Delft, 28 November, 2024
