# Supplementary material for: Prevention of postamputation pain with targeted muscle reinnervation (PreventPAP trial): protocol for a national, multicentre, randomised, sham-controlled trial
Source: BMJ Open. 2025 Nov 4;15(11):e105053. doi: 10.1136/bmjopen-2025-105053 (PMC12587972; doi:10.1136/bmjopen-2025-105053)
Supplement: online supplemental file 2 [file bmjopen-15-11-s002.pdf]

Supplementary file 2: Subject informed consent form

Prevention of PostAmputation Pain with Targeted Muscle Reinnervation (PreventPAP trial).

- I have read the information sheet. I was able to ask questions. My questions have been answered well enough. I had enough time to decide if I want to participate.
- I know that participation is voluntary. I also know that I can decide at any given time not to participate or to quit the study. I don't have to explain why.
- I give the researchers consent to inform my general practitioner that I participate in this trial.
- I give the researchers consent to request information from the general practitioner about for example my medical history or medication use.
- I give the researchers consent to request medication information from my pharmacy at the end of the study for a period of 15 months.
- I give the researchers consent to collect and use my data to answer the research question of this study.
- I give the researchers consent to store my data for 15 years in the hospital according to the European good clinical practice directive.
- I know that for the monitoring of this research some people can get access to all my data. These people are listed in the information sheet. I give consent for access by these people.

- I give consent to store my data for 15 years and to use it for further research in the field of my condition and/or the investigated treatment method, as stated in the information sheet.
  - **Yes**
  - **No**
- I give consent to ask me after this study if I want to participate in other research.
  - **Yes**
  - **No**
- I give consent to inform me after the study in which group I was randomized.
  - **Yes**
  - **No**

- I want to participate in this study.

My name is (subject): ………………………………..

Signature: ……………………… Date : __ / __ / __

I declare that I have fully informed this subject about the above study.

If any information becomes known during the study that could influence the subject's consent, I will let this subject know in good time.

Investigator name (or their representative):………………………………

Signature:………………………   Date: __ / __ / __

Additional information was given by:

Name:………………………………..

Job title:………………………………

Signature:………………………   Date: __ / __ / __

The subject will receive the complete information sheet, together with a signed version of the consent form.
