# Supplementary material for: Prevention of postamputation pain with targeted muscle reinnervation (PreventPAP trial): protocol for a national, multicentre, randomised, sham-controlled trial
Source: BMJ Open. 2025 Nov 4;15(11):e105053. doi: 10.1136/bmjopen-2025-105053 (PMC12587972; doi:10.1136/bmjopen-2025-105053)
Supplement: online supplemental file 3 [file bmjopen-15-11-s003.pdf]

Supplementary file 3. PreventPAP trial treatment protocol

All amputations in the PreventPAP trial should follow the steps below.

SURGICAL PROTOCOL

***in black: procedure for amputation only ('daily practice')**

Technical instructions on nerve handling in the control group:

- Neurectomy should be performed for:
    - Transtibial amputation: superfical peroneal nerve, deep peroneal nerve, tibial nerve, and saphenous nerve;
    - Transfemoral amputation and disarticulation: Saphenous nerve and sciatic nerve.
- Perform neurectomy (with or without traction) with scalpel (larger nerves) or scissors (smaller nerves); no ligation of nerve, no crush lesioning, no injection other than local anesthesia in interfascicular tissue, refrain from coagulation of bleeding nerve stump if possible, use collagen or similar to stop bleeding.
- To prevent kinking of the amputated nerves, reduce the length of the nerve to similar length as in the TMR group;

***in red and underlined: extra surgical steps for TMR treatment group**

TMR technical instructions:

- See for target nerves table below
- Perform nerve-nerve coaptation using 6-0 to 8-0 sutures, reinforce coaptation with fibrin glue;
- Avoid a long, winding, proximal nerve (risk of kinking!)
- Avoid superficial position of the coaptation (protection of coaptation)
- Please list Nerve Coaptation Caliber Mismatch Score 1-4 for all coaptations in your surgical report (see below)

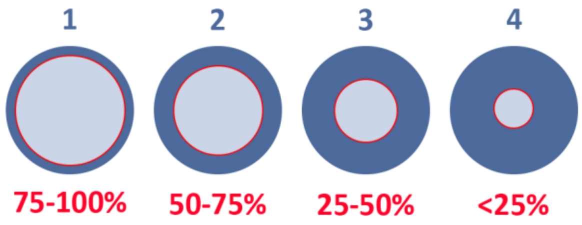

Nerve Coaptation Caliber Mismatch Score

TRANSTIBIAL- PROCEDURE STEP by STEP

1. Transtibial amputation following standard procedure:
    a. Skin incision according to Burgess' technique:
        - Start with longitudinal medial skin incision, identify saphenous nerve antero-medially;

        - Make anterior, lateral and posterior skin incisions, perform neurectomy of sural nerve at 2cm from distal end of the posterior flap
        - NB If needed extend both lateral and medial incisions (“fishmouth”) to get to target motor branches for saphenous and peroneal nerves in your surgical field;
    b. Identify the superficial (SPN) and deep peroneal (DPN) nerves anterior to the fibula (if needed follow proximal to fibular head to common peroneal nerve), then divide lateral compartment muscles, SPN and DPN neurectomy with preservation of length of SPN and DPN;
    c. Expose and divide tibia approximately 8-10cm from the tibial tubercle with an anterior bevel proximally;
    d. Expose and divide fibula 1 to 2 cm proximal to the residual tibia;
    e. Incision of the posterior compartment through the posterior intermuscular fascia, identify tibial nerve, neurectomy with preservation of length;
    f. The major vessels of the leg are ligated and divided when encountered;
    g. Resect distal portion of soleus muscle to reduce volume (if needed) to facilitate tension free closure and simplify TMR nerve coaptation;
    h. Remove tourniquet (if used), hemostasis;
2. Identify target motor nerve branches, cut distally.
    a. Coaptation with 8-0/6-0 epineural stitches, add collagen glue (table 1):
        i. SPN to motor branch m peroneus longus/brevis
        ii. DPN to motor branch m tibialis anterior
        iii. Tibial nerve to motor branch medial or lateral gastrocnemius
        iv. Saphenous nerve to motor branch medial gastrocnemius;
3. Myodesis of gastrocnemius muscle anteriorly over the distal tibia. Again, resect part of soleus muscle if needed;
4. Closure of fascia and skin.

TRANSFEMORAL - PROCEDURE STEP by STEP

1. Transfemoral amputation or disarticulation following standard procedure:
    a. Make ‘fishmouth’ skin incision;
    b. The quadriceps is detached just proximal to the patella, retaining some of its tendinous portion. The vastus medialis is reflected off the intermuscular septum.
    c. The adductor magnus is detached from the adductor tubercle by sharp dissection and reflected medially to expose the femoral shaft. It may be necessary to detach an additional 2 to 3 cm of adductor magnus from the linea aspera;
    d. Identify saphenous nerve under sartorius fascia, neurectomy with preservation of length;
    e. Identify motor branch sartorius muscle
    f. Identify vessels and ligated at or proximal to Hunter's canal. The smaller muscles (mm gracilis, sartorius, semimembranosus, and semitendinosus) may be transected approximately 2-5 cm longer than the proposed bone cut to facilitate their inclusion and anchorage as part of a myoplasty;

g. TMR of distal saphenous nerve to motor branch of m sartorius using 6-0/8-0 epineural stitches and collagen glue;
h. The femur is exposed approximately 12 to 14 cm above the condylar level and transected.
i. Muscles are sutured together by fascia approximating sutures;
j. (OPTIONAL: Two or three small drill holes are made on the lateral cortex of the distal femur and additional holes are made anteriorly and posteriorly approximately 1 to 1.5 cm from the cut end. The adductor magnus tendon then is sutured to the lateral aspect of the femur via the drill holes. Before securing the sutures, the femur is held in maximum adduction, while the adductor magnus is brought across the cut end of the femur, maintaining its tension. Additional anterior and posterior sutures are placed to prevent the muscle from sliding forward or backward over the end of the bone);
k. Remove tourniquet (if used), hemostasis. Closure of amputation wound.

Turn patient to Prone position

2. 10 cm incision midportion of the thigh starting as high as two fingers from the gluteal skin crease; make sure not to damage the posterior femoral cutaneous nerve in the subcutis;
   a. Open the fascia of the posterior compartment and identify the sciatic nerve, retract the cut nerve through the incision;
   b. Identify 4 motor branches (semitendinosus, semimembranosus muscles, 2 biceps femoris);
   c. Divide sciatic nerve in tibial and peroneal branch; Divide both nerves in two to reduce diameter, resulting in 4 fascicles of approx. 2-4cm for TMR;
   d. Tension free coaptation with 6-0/8-0 epineural stitches and fibrin glue
      i. 2 tibial components to two motor branches of the mm. semitendinosus and semimembranosus
      ii. 2 peroneal components of the sciatic nerve to the m. biceps femoris.
   e. Closure of the wound.

Nerve Transfers during Amputation: treated nerve and targets

Above knee	
Saphenous nerve	Sartorius
Sciatic nerve - tibial	1 Semitendinosus, 1 semimembranosus
Sciatic nerve- peroneal	biceps femoris (2 branches)
Below knee	
Tibial nerve	Medial or lateral gastrocnemius; (alternative: tibialis posterior; medial or lateral soleus)
Deep peroneal nerve	Tibialis anterior (alternative: peroneus longus; peroneus brevis; medial soleus)
Superficial peroneal nerve	Peroneus longus or peroneus brevis
Saphenous nerve	Medial gastrocnemius (alternative: medial soleus; vastus medialis)

REMARKS

- Note that in the transfemoral control group a cutaneous sham incision is made of 5-10cm; Make sure identical closure technique and dressing is used as in the treatment group.
- During surgery, patients will receive general anesthesia. Epidural anesthesia is not allowed in the study protocol, as nerve conduction can be reduced, and stimulation is not reliable.
- If a tourniquet is used to perform the amputation, maximal time of insufflation is 60 minutes, after which intraoperative nerve stimulation becomes less reliable due to tourniquet neurapraxia.
- Avoid atrophy and loss of soft tissue cushioning over the bony prominences.
- In case of severe size mismatches (see mismatch score above) separate the nerve fascicles into smaller units for multiple transfers.

PAIN MANAGEMENT

- **Preoperative epidural of peripheral nerve blocks are not allowed in the study as it interferes with the TMR technique.**
- The choice of anesthetics used will depend on the preference of the anesthesiologist. Post-operative analgesia is achieved using the post-operative analgesia protocol of the hospital where the operation is performed. Paracetamol, NSAIDs and weak and/or strong opiates will be used for this.
- Neuropathic pain medication, such as pregabalin, may be considered.
- For the treatment of postoperative pain, a postoperative epidural or peripheral block and catheter can be used;

POSTOP PROTOCOL

- Early shrinker wear after removal of drains (e.g. Ossur Rigid dressing)
- The prosthetic may be applied after 2 weeks postoperatively, provided the wound has healed sufficiently.
- Start weight-bearing after 2 weeks postoperatively, provided the wound has healed sufficiently.
