# Supplementary material for: Prevention of postamputation pain with targeted muscle reinnervation (PreventPAP trial): protocol for a national, multicentre, randomised, sham-controlled trial
Source: BMJ Open. 2025 Nov 4;15(11):e105053. doi: 10.1136/bmjopen-2025-105053 (PMC12587972; doi:10.1136/bmjopen-2025-105053)
Supplement: Supplementary file 4 [file bmjopen-2025-105053.reviewer_comments.pdf]

PEER REVIEW HISTORY

BMJ Open publishes all reviews undertaken for accepted manuscripts. Reviewers are asked to complete a checklist review form and are provided with free text boxes to elaborate on their assessment. These free text comments are reproduced below.

ARTICLE DETAILS

Title (Provisional)

Prevention of PostAmputation Pain with Targeted Muscle Reinnervation (PreventPAP trial): protocol for a national, multicenter, randomized, sham-controlled trial

Authors

Tendijck, Guus A H; van Schaik, Jan; Dijkman, Robert R; Niesters, Marieke; van Zwet, Erik W; van den Hout, Wilbert B; Ploeg, Arianne J; van Rijt, Willem G; de Ruiter, Godard; Coert, J. Henk; Duraku, Liron S; Zuidam, Jelle; van de Water, Willemien; Pondaag, Willem; van der Krogt, Hanneke; Groen, Justus L; consortium, PreventPAP

VERSION 1 - REVIEW

**Reviewer**	**1**
**Name**	**Mayo, Amanda**
**Affiliation**	**Sunnybrook Health Sciences Centre**
**Date**	**04-Aug-2025**
**COI**	**None**

Very important area of study in limb loss population, innovative in vascular population. Minor revisions suggested

Line 41 - first primary TMR study in non-trauma patients, I believe Duhamian et al have published in trauma limb loss population as well as Jana Friedly's group in Seattle Washington both published in 2014

Line 63-64: Residual limb pain is not mostly driven by neuromas - other causes include infection/heterotopic ossification/adherent scar tissue etc etc revise statements re: residual limb pain and mention neuromas are a portion of cause, in my experience ~1/3 due to symptomatic neuromas and I believe studies support this

Line 131 - Define physiologically unstable unclear what this means

---

**Reviewer**	**2**

**Name**	**Xu, Simao**
**Affiliation**	**Chengdu Sport University**
**Date**	**05-Sep-2025**
**COI**	**None**

Approximately 2200 major lower extremity amputations, transfemoral to transtibial, are performed in the Netherlands each year, and the incidence is rising.1, 2 Around 61% of these patients develop postamputation pain (PAP).3 PAP is known to compromise prosthetic rehabilitation and profoundly diminishes the quality of life after amputation.4 Patients with chronic pain report a lower health related quality of life when a neuropathic component is present (0.70 versus 0.47).5 Moreover, it is associated with a significant burden on the healthcare system.6 Direct healthcare costs may comprise pre-hospital care, emergency care, hospitalization, long-term post-discharge care, and rehabilitation (e.g.increased opioid use and other (neuropathic) pain medication and interventions by pain specialists to treat PAP). Indirect costs, may arise as a consequence of loss of productivity for both patients and family.

Based on this, design “Prevention of PostAmputation Pain with Targeted Muscle Reinnervation (PreventPAP trial): protocol for a national, multicenter, randomized, sham-controlled trial”.This topic has certain research value, with clear research ideas and a reasonable research plan.

Q:The reasons for proposing PTM are not sufficient, and the innovation is not reflected sufficiently.

Q:The sample size required for the study lacks necessary calculation explanations.

Q:The description of statistical methods is incomplete.

VERSION 1 - AUTHOR RESPONSE

Reviewer: 1
Dr. Amanda Mayo, Sunnybrook Health Sciences Centre
Comments to the Author:

Line 41 - first primary TMR study in non-trauma patients, I believe Duhamian et al have published in trauma limb loss population as well as Jana Friedly's group in Seattle Washington both published in 2014

Line 41 refers to the bullet-point in the strengths and limitation section ‘This is the first trial to evaluate primary targeted muscle reinnervation (TMR) during amputations as a prophylactic strategy for pain.’ After further reconsideration, we decided to removed this

bullet point from the list, because it was not in line with the journal's policies. The journal, requires bullet points in the strength and limitation section, that specifically relate to the methods. Because this sentence could be considered a novelty, we were required by the journal's policy to change or remove this statement. Therefore, we decided to replace this bullet point.

Line 63-64: Residual limb pain is not mostly driven by neuromas - other causes include infection/heterotopic ossification/adherent scar tissue etc etc revise statements re: residual limb pain and mention neuromas are a portion of cause, in my experience ~1/3 due to symptomatic neuromas and I believe studies support this

We agreed with your suggestion and changed the text in the manuscript. We decided not to report the proportions of symptomatic neuromas as a cause for residual limb pain. Mainly because in the meta-analyses that describe the prevalance of symptomatic neuromas (List et al.(1) and Huang et al.(2)), the included studies are of low quality, hetrogeneous, and it is unclear how the diagnosis of symptomatic neuromas was established.

Line 131 - Define physiologically unstable unclear what this means

Line 131 referred to an exclusion criterium: 'physiologically unstable patients at the time of amputation'. Upon reviewing the exclusion criterium, we recognize the term may have been vague. In this context, by 'physiologically unstable', we meant patients requiring immediate amputation due to systemic decompensation or multiorgan failure (e.g. due to a severe infection causing sepsis). These patients may not have enough time to consent for this study, be temporarily cognitive impaired that prevents the patient from understanding the informed consent procedure, or may be rejected by the anesthesiologist due to their critical condition. Because, these variables were already specified in the protocol, the exclusion criterium was deemed redundant. Therefore, we felt it was more appropriated to remove this exclusion criterium from the protocol.

Reviewer: 2
Prof. Simao Xu, Chengdu Sport University
Comments to the Author:

Q:The reasons for proposing PTM are not sufficient, and the innovation is not reflected sufficiently.

We are not familiar with term PTM, could you please clarify?

Q:The sample size required for the study lacks necessary calculation explanations.

Based on the study of Farrar et al. a change in the numeric rating scale (NRS) of two points has been shown to be clinically relevant and correlated to a patient's need to take additional pain medication.(3) To detect a minimal clinically important difference of two points on the NRS, we used a standard deviation of 3.5. This standard deviation was based on similar studies that measured pain at one timepoint after 12 months (Berger et al.(4), O'Brien et al.(5), Mioton et al.(6), and Kang et al.(7)).
To account for multiple comparisons we will test RLP and PLP at significance level 0.025 (two-sided) using the Bonferroni correction. To achieve 90% power, with a difference of 2 NRS points with a standard deviation of 3.5, and a p-value of 0.025, we need to analyze 152 patients. This is based on the standard formula to calculate the sample size in medical studies see below:

Given:
$\Delta = |\mu 2-\mu 1|$ = absolute difference between two means (2)
$\sigma 1, \sigma 2$ = variance of mean #1 and #2 (3.5)
n1 = sample size for group #1
n2 = sample size for group #2
α = probability of type I error (0.025)
β = probability of type II error (0.10)
z = critical Z value for a given α or β
k = ratio of sample size for group #2 to group #1 (1)

k = n2/n1 = 1
n1=(σ1^2 + σ2^2)(z(1-α/2)+z(1-β))^2/Δ^2
n1=(3.5^2 + 3.5^2)(2.24+1.28)^2/2^2 = 75.89

Because both groups are compared in a ratio of 1:1, the total sample size is 76x2 = 152 patients.
To include enough patients to achieve the sample size of 152 patients we estimated a loss to follow up of 6%. Resulting in 161.12 patients, which equals to 162 patients. Moreover we estimate the 1 year mortality of our patient population to be around 20%, based on the study of Cascini et al.(8) and our clinical experience. Which means we have to include 202.5 = 203 patients to be able to randomize 152 patients. This has been clarified in the protocol.

Q:The description of statistical methods is incomplete.

Upon further consideration, we recognized the need to clarify our statistical approach. Given that we will evaluate the same data at multiple timepoints, we have replaced multiple imputation with a repeated measure analysis. This change will better account for missing data and provide more precise and unbiased estimates in patients where the same variables are

measured over time.
In the primary study parameters we clarified the gatekeepers procedure to first assess the PROMIS interference short form and second the PROMIS behavior short form. We also removed redundant text form the secondary study parameters for clarification purposes.

References
1. List EB, Krijgh DD, Martin E, et al. Prevalence of residual limb pain and symptomatic neuromas after lower extremity amputation: a systematic review and meta-analysis. Pain. 2021;162(7):1906–13.
2. Huang YJ, Assi PE, Drolet BC, et al. A Systematic Review and Meta-analysis on the Incidence of Patients With Lower-Limb Amputations Who Developed Symptomatic Neuromata in the Residual Limb. Ann Plast Surg. 2022;88(5):574–80.
3. Farrar JT, Young JP, Jr., LaMoreaux L, et al. Clinical importance of changes in chronic pain intensity measured on an 11-point numerical pain rating scale. Pain. 2001;94(2):149–58.
4. Berger LE, Shin S, Haffner ZK, et al. The application of targeted muscle reinnervation in lower extremity amputations: A systematic review. Microsurgery. 2023;43(7):736–47.
5. O'Brien AL, West JM, Gokun Y, et al. Longitudinal Durability of Patient-Reported Pain Outcomes after Targeted Muscle Reinnervation at the Time of Major Limb Amputation. J Am Coll Surg. 2022;234(5):883–9.
6. Mioton LM, Dumanian GA, Shah N, et al. Targeted Muscle Reinnervation Improves Residual Limb Pain, Phantom Limb Pain, and Limb Function: A Prospective Study of 33 Major Limb Amputees. Clin Orthop Relat Res. 2020;478(9):2161–7.
7. Kang NV, Woollard A, Michno DA, et al. A consecutive series of targeted muscle reinnervation (TMR) cases for relief of neuroma and phantom limb pain: UK perspective. J Plast Reconstr Aesthet Surg. 2022;75(3):960–9.
8. Cascini S, Agabiti N, Davoli M, et al. Survival and factors predicting mortality after major and minor lower-extremity amputations among patients with diabetes: a population-based study using health information systems. BMJ Open Diabetes Res Care. 2020;8(1).
